# The impact of a multi-domain intervention on cerebral glucose metabolism: analysis from the randomized ancillary FDG PET MAPT trial

**DOI:** 10.1186/s13195-020-00683-6

**Published:** 2020-10-19

**Authors:** Julien Delrieu, Thierry Voisin, Laure Saint-Aubert, Isabelle Carrie, Christelle Cantet, Bruno Vellas, Pierre Payoux, Sandrine Andrieu

**Affiliations:** 1 Pôle gériatrie, Cité de la santé, Place Lange - TSA 60033, 31059 Toulouse Cedex 9, France; 2 INSERM UMR 1027, Toulouse, France; University of Toulouse III, Toulouse, France; 3 Gérontopôle, Department of Geriatrics, Toulouse (University Hospital) CHU, Purpan University Hospital, Toulouse, France; 4 Toulouse NeuroImaging Center, University of Toulouse III, INSERM, UPS, Toulouse, France; 5 Department of Nuclear Medicine, Toulouse CHU, Purpan University Hospital, Toulouse, France; 6 Toulouse NeuroImaging Center, University of Toulouse, INSERM, UPS, Toulouse, France; 7 Department of Epidemiology and Public Health, Toulouse CHU, Toulouse, France

**Keywords:** Clinical trials randomized controlled, All cognitive disorders/dementia, Alzheimer’s disease, PET, Prevention

## Abstract

**Background:**

The Multidomain Alzheimer Preventive Trial (MAPT) was designed to assess the efficacy of omega-3 fatty acid supplementation, multidomain intervention (MI), or a combination of both on cognition. Although the MAPT study was negative, an effect of MI in maintaining cognitive functions compared to placebo group was showed in positive amyloid subjects. A FDG PET study (MAPT-NI) was implemented to test the impact of MI on brain glucose metabolism.

**Methods:**

MAPT-NI was a randomized, controlled parallel-group single-center study, exploring the effect of MI on brain glucose metabolism. Participants were non-demented and had memory complaints, limitation in one instrumental activity of daily living, or slow gait. Participants were randomly assigned (1:1) to “MI group” or “No MI group.” The MI consisted of group sessions focusing on 3 domains: cognitive stimulation, physical activity, nutrition, and a preventive consultation. [^18^F]FDG PET scans were performed at baseline, 6 months, and 12 months, and cerebral magnetic resonance imaging scans at baseline. The primary objective was to evaluate the MI effect on brain glucose metabolism assessed by [^18^F]FDG PET imaging at 6 months. The primary outcome was the quantification of regional metabolism rate for glucose in cerebral regions involved early in Alzheimer disease by relative semi-quantitative SUVr (FDG-based AD biomarker). An exploratory voxel-wise analysis was performed to assess the effect of MI on brain glucose metabolism without anatomical hypothesis.

**Results:**

The intention-to-treat population included 67 subjects (34 in the MI group and 33 in the No MI group. No significant MI effect was observed on primary outcome at 6 months. In the exploratory voxel-wise analysis, we observed a difference in favor of MI group on the change of cerebral glucose metabolism in limbic lobe (right hippocampus, right posterior cingulate, left posterior parahippocampal gyrus) at 6 months.

**Conclusions:**

MI failed to show an effect on metabolism in FDG-based AD biomarker, but exploratory analysis suggested positive effect on limbic system metabolism. This finding could suggest a delay effect of MI on AD progression.

**Trial registration:**

ClinicalTrials.gov Identifier, NCT01513252.

## Background

The MAPT (Multidomain Alzheimer Prevention Trial) study has tested the effect of omega 3 polyunsaturated fatty acid supplementation (omega-3) and multidomain intervention (MI), alone or in combination, on cognitive decline in elderly people with memory complaint [1]. The MI and omega-3, alone or in combination, had no significant effect on cognitive decline over 3 years [2]. However, the ancillary amyloid MAPT study (MAPT-AV45) has provided new insights on the effects of MI alone or in combination with omega-3 in positive amyloid subjects in maintaining cognitive functions compared to the placebo group [3]. Thus, a MI effect can be suspected at early stages of AD.

The implementation of AD biomarkers in prevention trials has become increasingly important to explore MI effect on cognition in a specific sub-population [3], and as outcome to better understand its mechanism of action [4]. To investigate the presumed effect of MI in early AD, we designed the MAPT-NeuroImaging (MAPT-NI) study, a randomized controlled parallel-group monocentric study, exploring the MI effect on glucose metabolism, in particular in cerebral areas involved early in neural dysfunction of AD. Metabolism by positron emission tomography with fluorine-18 fluorodeoxyglucose ([^18^F]FDG PET) is considered as an AD biomarker [5] and a potential surrogate marker of AD progression [6]. Indeed, cerebral metabolism is proposed as biomarker of “neuronal injury” in the revised criteria proposed by the national institute of aging and Alzheimer association (NIA-AA) and as biomarker of “progression” in the research criteria proposed by the international working group-2 [7, 8]. Progressive decrease of metabolism in associative cortices is closely related to progressive cognitive impairment and allows monitoring of disease progression not provided by pathophysiological biomarkers [9]. Brain metabolism measurements may be used in clinical trials as endpoint to better understand the mechanisms of action of an intervention [6, 10, 11]. The number of published trials using [^18^F]FDG PET as outcome is limited, and in large MI trials (Prevention of Dementia by Intensive Vascular Care, Finnish Geriatric Intervention Study to Prevent Cognitive Impairment and Disability, FINGER) [12, 13], glucose metabolism has never been—to date—used for this purpose. The MAPT-NI study is a unique opportunity to assess the impact of a MI, alone or in combination with omega-3, on metabolism and to explore their potential mechanism of action on cognitive performance. We hypothesize that the MI, in non-demented subjects, alone or in combination with omega-3, positively affects metabolism in regions early involved in AD after 6-month intervention.

## Methods

### Study design and participants

All subjects enrolled in this ancillary [^18^F]FDG PET study were participants from the MAPT study. The MAPT protocol is registered on a public-access clinical trial database (www.clinicaltrials.gov, no. NCT01513252). MAPT-NI study protocol was approved by the French Ethics Committee in Toulouse and AFSSAPS (national agency for the safety of drugs and health products) in February 2009. One hundred seventy-eight subjects were asked consecutively to participate to MAPT-NI at their inclusion visit in MAPT, and 68 subjects have been included. Written informed consent was given by all participants. Included subjects were 70 years old and over and fulfilled at least one of the following three clinical criteria: spontaneous memory complaint, limitation in one instrumental activity of daily living, or slow gait. Subjects with dementia were not included in this trial.

[^18^F]FDG PET scans were performed at baseline (within 1 month following written consent), 6 months, and 12 months, and cerebral magnetic resonance imaging (MRI) scans at baseline. Baseline MRI scans were designed to detect significant exclusion abnormalities (surgical lesions and significant cerebrovascular lesions) and to assess medial temporal lobe atrophy using a 0–4 rating scale [14]. APOE genotyping was determined from blood samples collected and stored at baseline for RNA/DNA extraction.

### Randomization and masking

In MAPT trial, participants were randomly assigned (1:1:1:1) to one of the four following groups: “MI plus omega-3”, “MI only”, “Omega-3 only”, and “Placebo only”. At the same time in MAPT-NI ancillary study, participants were randomly assigned (1:1) to MI group or No MI group. However, MAP-NI participants of MI group and No MI group were also allocated to omega-3 or placebo. At the end of the MAPT trial, the distribution of the subjects between the 4 interventional groups was only known after unblinding. All participants, neuropsychologists, and research staff were blinded to omega-3 or placebo assignment and to [^18^F]FDG PET assessment.

### Procedures

#### Multi-domain intervention and omega-3 supplementation

The MI consisted of group sessions focusing on 3 domains: cognitive stimulation, physical activity, nutrition, and a preventive consultation as described previously [1, 2]. Briefly, each session included 60 min of cognitive training, 45 min of demonstrations about physical activity, and 15 min of nutritional advice. Participants with MI underwent 12 sessions of 2 h in the first 2 months, followed by a 1-h session once a month, and finally a 2-h session at 12 months. The active supplement used was V0137, an oil mixture containing natural fish oil with a minimum of 65% docosahexaenoic acid (DHA) and a maximum of 15% eicosapentaenoic acid (EPA). Participants took two capsules daily of either the supplement or the placebo.

#### Cognitive assessment

Clinical visits were scheduled at baseline, 6 months, and 12 months to assess physical and cognitive performances and adherence. A comprehensive assessment of cognitive functions was performed, including the Free and Cued Selective Reminding Test (FCRST) [15], the Controlled Oral Word Association Test and Category Naming Test (COWAT and CNT) [16], the Digit Symbol Substitution Subtest of the Wechsler Adult Intelligence Scale–Revised [17], the Trail-Making Test (TMT) [18], the Mini-Mental State Examination (MMSE) [19], and the Clinical Dementia Rating (CDR) [20]. A cognitive composite score was calculated at each time point (baseline, 6 months, 12 months) by averaging the standardized *Z* scores at four cognitive tests (FCRST, CNT, Digit Symbol Substitution test, orientation items of MMSE) [21].

#### [^18^F]FDG PET and MRI exams

[^18^F]FDG PET scans were acquired at the Toulouse Hospital PET center, on a Biograph™ 6 TruePoint™ (Siemens Medical Solutions, Knoxville, TN USA) high-resolution PET/CT scanner (3D detection mode, producing images with 1 × 1 × 1.5-mm voxels and a spatial resolution of 5 mm full width at half maximum at the field of view center), during 20 min in list mode, 30 min after injection of 1.85 MBq/kg weight of [^18^F]FDG on average (± 10%). All images benefited from a partial volume effect correction on this machine. Plasma glucose levels of all MAPT-NI participants have been checked before FDG PET scans with a fast of at least 4 h (glucose threshold < 10 mmol/l). For each patient, [^18^F]FDG PET scans from all time points were first realigned onto their mean image and normalized using the same transformation matrix in the MNI space using a PET template in Statistical Parametric Mapping 12 (SPM12) software running on Matlab. Cortical standardized uptake value ratio (SUVr) images were obtained using the gray matter of the cerebellum as reference region. Regions of interest (ROIs) involved early in AD were selected based on the MetaROI approach described by Landau et al. [22]. [^18^F]FDG mean SUVr uptake was quantified in 6 predefined cortical ROIs, extracted from a cortical atlas derived from the Harvard-Oxford atlas (FSL software, The University of Oxford): right and left posterior cingulate, angular gyrus, and middle/inferior temporal areas.

The MRI scans were performed at baseline visit using a standardized protocol including these sequences: 3D T1-weighted, T2 FLAIR, T2 TSE, and T2 GRE. A local independent radiologist assessed MRI scans to detect significant exclusion abnormalities.

#### Adherence

For supplementation, adherence was assessed by counting the number of capsules returned by participants. For the MI, adherence was calculated as the percentage of intervention sessions attended. Participants were deemed adherent if they took at least than 75% of the prescribed capsules and attended at least 75% of the MI group sessions (if applicable).

### Objectives and outcomes

#### Primary objective

The primary objective was to evaluate the MI effect on brain glucose metabolism assessed by [^18^F]FDG PET imaging at 6 months. The primary outcome was the quantification of regional metabolism rate for glucose by relative semi-quantitative SUVr. This global SUVr value (AD-based SUVr) was used as an FDG-based AD biomarker and primary outcome (supplementary Fig. 1).

#### Secondary and exploratory objectives

The secondary objectives were (1) to assess the long-term effect of MI on AD-based SUVr at 12 months and (2) to test the effect of omega-3 supplementation on AD-based SUVr at 6 and 12 months.

The exploratory objectives were (1) to assess the effect of combination of MI and omega-3 supplementation on AD-based SUVr; (2) to explore, using a voxel-wise approach, the effect of interventions on metabolism; (3) to test the effect of MI and omega-3 supplementation according adherence; and (4) to explore effect of interventions on cognitive composite score.

### Sample size

The number of participants required was calculated referring to study of De Leon et al. [23]. We expected a 5% increase on cerebral glucose metabolism in the MI group and no progression in the No MI group. For a power of 90% for bilateral alpha risks of 0.05 (in the case of 10 comparisons performed), the sample size to be recruited was 34 subjects per group or 68 subjects for the entire study (*n* = 1680).

### Statistical analysis

#### Baseline demographic analysis

We compared baseline characteristics of subjects according to their group intervention: (1) “MI group” vs. “No MI group”, (2) “Omega-3 group” vs “No omega-3 group”, and (3) “MI only group” vs “Omega-3 only group” vs “MI plus omega-3 group” vs “Control group”. We used *χ*^2^ or Fisher’s exact (for expected values < .05) tests for categorical variables, one-way analyses of variance for quantitative variables with normal distributions (Student’s tests or Fisher’s tests), and non-parametric tests (Kruskal-Wallis test) for quantitative variables without normal distributions.

#### ROI-based approach

Analysis was conducted in the intention-to-treat (ITT, *n* = 67, primary analysis) population and a sub-sample with adherence (exploratory analysis). ITT population included all randomly assigned participants who completed at least one [^18^F]FDG PET scan at baseline, 6 months, or 12 months. In the sub-sample with adherence analysis, participants were deemed adherent if they attended at least 75% of MI group sessions (*n* = 20) and took at least 75% of the prescribed capsules (*n* = 30).

Linear mixed-model repeated-measures analyses were applied to baseline, 6-month, and 12-month data to assess between-group differences in the change on AD-based SUVr along time. Time was used as a continuous variable. All the models were completed with and without adjustments for gender, age, level of education, global CDR score, APOE-4 genotype, and group intervention. For each linear mixed model, we included subject-specific random effects to take into account the intra-subject correlation: a random intercept to take into account the heterogeneity of the AD-based SUVr at baseline and a random slope to take into account the heterogeneity of the slopes between subjects if this parameter was significant. In the unadjusted linear mixed models, we included the following fixed effects: intervention group, time, and interaction between group and time. All confidence intervals (CIs) were two-sided with a 95% confidence level, and the statistical significance was set at a *p* value < .05. All statistical analyses were performed using SAS software version 9.4 (SAS Institute Inc., Cary, NC).

#### Voxel-wise approach

To investigate potential group differences on metabolic changes without anatomical a priori, we performed voxel-wise analyses on smoothed (8 × 8 × 8) [^18^F]FDG SUVr images using SPM12. To address the effect of MI and omega-3 along time, voxel-wise *t* tests were performed to assess change on glucose metabolism from baseline to 6 months and 12 months with groups defined as MI group vs No MI group and Omega-3 vs No omega-3. [^18^F]FDG SUVr images were smoothed (8 × 8 × 8) and delta images (6 months-baseline, 12 months-6 months, and 12 months-baseline) were created for each subject. Voxel-wise differences on changes in glucose metabolism from baseline to 6 and 12 months between the 4 randomized groups were assessed using a one-way ANOVA. A peak threshold of *p* = .001 (uncorrected, and also Family Wise Error Rate < .05) with an extent threshold of *k* = 50 voxels for significant clusters was chosen. The MNI coordinates of the local maxima in each significant cluster were then reported onto the Harvard-Oxford atlas (FSL software, The University of Oxford) for regional labeling.

## Results

### Enrollment and rates of study completion

**Fig. 1:**
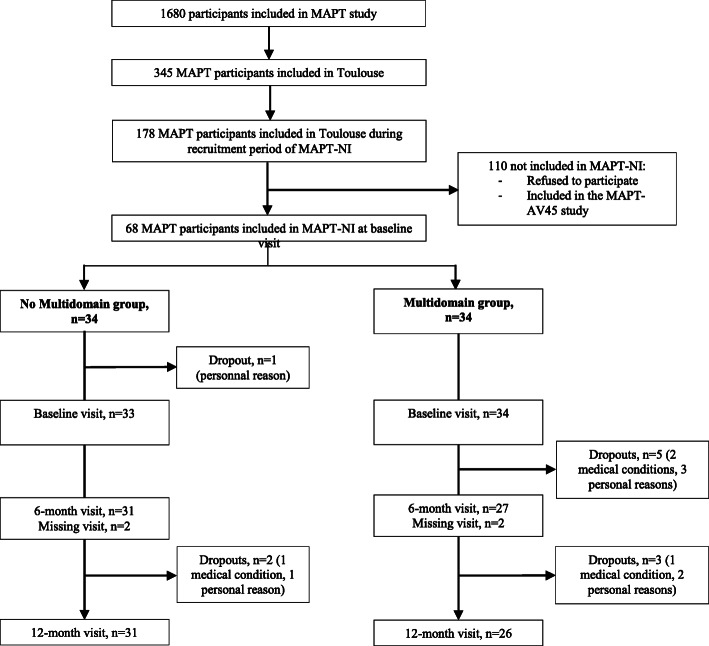
Trial profile of the MAPT-NI study. Abbreviations: MAPT, Multidomain Prevention Alzheimer Trial; MAPT-NI, MAPT-NeuroImaging; PET, positron emission tomography; MI, multidomain intervention

Of the 345 participants from MAPT recruited at Toulouse center, 68 subjects were included in MAPT-NI. Sixty-seven FDG PET scans were performed in the MAPT-NI study at baseline, 58 and 57 respectively at 6- and 12-month visits. Subjects were enrolled between May 6, 2009, and February 9, 2011. At baseline, spontaneous memory complaint was present in 57 (85.07%) of the 67 participants and slow walking speed in 1 (1.49%). No participants were included only on limitation in one instrumental activity of daily living. Seven (10.46%) participants reported two of these factors, and 2 (2.99%) reported all three factors. The flow chart of MAPT-NI participants is showed in Fig. 1. The ITT population included 67 subjects (34 in the MI group and 33 in the No MI group). Twenty-six (76.47%) subjects of the MI group completed the follow-up and 31 (93.94%) of the No MI group.

### Baseline characteristics

Subjects who participated to MAPT-NI were significantly older (76.37 ± 4.23 vs 75.29 ± 4.43 years, *p* = .022) and had a lower Geriatric Depression Scale (GDS) score (2.33 ± 1.85 vs 3.31 ± 2.65, *p* = .005) than MAPT subjects non-included in MAPT-NI sub-study. Baseline characteristics of the 67 participants included in the ITT analysis are presented in the Table 1. The MI group and No MI group did not differ significantly for baseline characteristics. The Omega-3 group and No omega-3 group differed significantly for age (*p* = 0.035, Table 1). Groups did not differ for diabetes and blood glucose level before FDG PET scan at baseline visit.

**Table 1:** Baseline characteristics of MAPT-NI groups

	TEP-FDG (***n*** = 67)								
		Primary population		Secondary and exploratory populations					
	Overall population (*n* = 67)	No MI (*n* = 33)	MI (*n* = 34)	No omega-3 (*n* = 29)	Omega-3 (*n* = 38)	Omega-3 + MI (*n* = 20)	Omega-3 only (*n* = 18)	MI only (*n* = 14)	Placebo (*n* = 15)
**Male gender**, *N* (%)	18 (26.87)	8 (24.24)	10 (29.41)	8 (27.59)	10 (26.32)	7 (35.00)	3 (16.67)	3 (21.43)	5 (33.33)
**Age in years**, mean (SD)	76.37 (4.23)	76.79 (4.24)	75.97 (4.24)	75.41 (4.37)	77.11 (4.02)	77.10 (4.15)	77.11 (3.98)	74.36 (3.95)	76.40 (4.64)
**BMI (kg/m2),** mean (SD)	26.40 (3.51)	26.60 (3.97)	26.21 (3.06)	26.35 (2.99)	26.44 (3.91)	26.34 (3.73)	26.56 (4.20)	26.04 (1.84)	26.65 (3.82)
**Education**, *N* (%)									
No diploma or primary school certificate	18 (27.27)	9 (27.27)	9 (27.27)	6 (21.43)	12 (31.58)	7 (35.00)	5 (27.78)	2 (15.38)	4 (26.67)
Secondary education	21 (31.82)	11 (33.33)	10 (30.30)	7 (25.00)	14 (36.84)	6 (30.00)	8 (44.44)	4 (30.77)	3 (20.00)
High-school diploma	9 (13.64)	4 (12.12)	5 (15.15)	6 (21.43)	3 (7.89)	2 (10.00)	1 (5.56)	3 (23.08)	3 (20.00)
University level	18 (27.27)	9 (27.27)	9 (27.27)	9 (32.14)	9 (23.68)	5 (25.00)	4 (22.22)	4 (30.77)	5 (33.33)
**APOE4 carrier**, *N* (%)	8 (13.79)	4 (13.33)	4 (14.29)	5 (20.00)	3 (9.09)	2 (12.50)	1 (5.88)	2 (16.67)	3 (23.08)
**Composite cognitive score,** mean (SD)	0.05 (0.69)	− 0.02 (0.71)	0.11 (0.68)	0.08 (0.58)	0.02 (0.77)	0.02 (0.80)	0.03 (0.77)	0.24 (0.46)	− 0.07 (0.66)
**MMSE total score**/30, mean (SD)	28.19 (1.62)	28.21 (1.71)	28.18 (1.55)	28.45 (1.40)	28.00 (1.76)	28.20 (1.54)	27.78 (1.99)	28.14 (1.61)	28.73 (1.16)
**MMSE orientation score**/10, mean (SD)	9.84 (0.41)	9.82 (0.39)	9.85 (0.44)	9.83 (0.38)	9.84 (0.44)	9.85 (0.49)	9.83 (0.38)	9.86 (0.36)	9.80 (0.41)
**CDR score**, *N* (%)									
CDR = 0	27 (40.30)	14 (42.42)	13 (38.24)	12 (41.38)	15 (39.47)	8 (40.00)	7 (38.89)	5 (35.71)	7 (46.67)
CDR = 0.5	40 (59.70)	19 (57.58)	21 (61.76)	17 (58.62)	23 (60.53)	12 (60.00)	11 (61.11)	9 (64.29)	8 (53.33)
**FCSRT scores**, mean (SD)									
Free recall/48	28.30 (6.81)	27.67 (7.47)	28.91 (6.16)	28.79 (6.22)	27.92 (7.29)	27.90 (6.54)	27.94 (8.25)	30.36 (5.47)	27.33 (6.69)
Total recall/48	45.34 (3.45)	45.03 (3.23)	45.65 (3.68)	45.38 (3.00)	45.32 (3.79)	45.15 (4.25)	45.50 (3.33)	46.36 (2.65)	44.47 (3.11)
Delayed free recall/16	10.93 (2.66)	10.82 (3.00)	11.03 (2.33)	11.21 (2.06)	10.71 (3.06)	10.90 (2.55)	10.50 (3.60)	11.21 (2.04)	11.20 (2.14)
Delayed total recall/16	15.61 (0.85)	15.48 (1.06)	15.74 (0.57)	15.79 (0.49)	15.47 (1.03)	15.75 (0.55)	15.17 (1.34)	15.71 (0.61)	15.87 (0.35)
**TMT A**, mean (SD)	44.78 (13.95)	45.03 (15.38)	44.53 (12.63)	43.52 (11.11)	45.74 (15.85)	45.30 (13.59)	46.22 (18.44)	43.43 (11.53)	43.60 (11.11)
**TMT B**, mean (SD)	114.36 (36.35)	116.03 (41.27)	112.74 (31.48)	108.56 (35.06)	118.97 (37.20)	119.22 (29.85)	118.69 (45.11)	103.77 (32.63)	113.00 (37.84)
**Code test score**, mean (SD)	38.19 (9.20)	37.97 (8.88)	38.41 (9.63)	37.90 (8.89)	38.42 (9.55)	37.75 (9.69)	39.17 (9.61)	39.36 (9.83)	36.53 (8.01)
**COWAT score**, mean (SD)	19.66 (6.24)	19.18 6.59)	20.12 (5.94)	20.34 (6.34)	19.13 (6.20)	19.65 (6.39)	18.56 (6.10)	20.79 (5.38)	19.93 (7.28)
**CNT score**, mean (SD)	25.70 (8.53)	24.94 (8.62)	26.44 (8.51)	26.52 (7.82)	25.08 (9.09)	25.45 (9.00)	24.67 (9.44)	27.86 (7.86)	25.27 (7.83)
**ADCS-ADL PI** /45; mean (SD)	40.00 (4.72)	39.27 (5.43)	40.73 (3.83)	40.45 (4.54)	39.65 (4.89)	40.00 (4.16)	39.28 (5.65)	41.71 (3.22)	39.27 (5.34)
**GDS, mean (SD)**	2.33 (1.85)	2.67 (1.67)	2.00 (1.98)	2.41 (2.01)	2.26 (1.75)	1.90 (1.74)	2.67 (1.71)	2.14 (2.35)	2.67 (1.68)
**AD-based SUVr,** mean (SD)	1.13 (0.10)	1.12 (0.08)	1.14 (0.11)	1.12 (0.1)	1.14 (0.09)	1.14 (0.11)	1.13 (0.08)	1.13 (0.11)	1.11 (0.09)
**DHA** (μg/g RBC), mean (SD)	5.82 (1.41)	5.66 (1.39)	5.98 (1.43)	5.69 (1.24)	5.92 (1.55)	5.97 (1.75)	5.88 (1.36)	6.00 (0.94)	5.41 (1.44)
**Medial temporal lobe atrophy,** *N* (%)									
Stage 0	17 (25.37)	6 (18.18)	11 (32.35)	8 (27.59)	9 (23.68)	4 (20)	5 (27.78)	7 (50)	1 (6.67)
Stage 0.5	13 (19.40)	6 (18.18)	7 (20.59)	3 (10.34)	10 (26.32)	4 (20)	6 (33.33)	3 (21.43)	0 (0)
Stage 1	25 (37.31)	14 (42.42)	11 (32.35)	13 (44.83)	12 (31.58)	8 (40)	4 (22.22)	3 (21.43)	10 (66.67)
Stage 1.5	5 (7.46)	3 (0.09)	2 (5.88)	3 (10.34)	2 (5.26)	1 (5)	1 (5.56)	1 (7.14)	2 (13.33)
Stage 2	7 (10.44)	4 (12.12)	3 (8.82)	2 (6.90)	5 (13.16)	3 (15)	2 (11.11)	0 (0)	2 (13.33)

Medial temporal lobe atrophy is the average of the left and right medial temporal lobes atrophy

*Abbreviations: MMSE* Mini-Mental State Examination, *CDR* Clinical Dementia Rating score, *ADCS-ADL PI* Alzheimer’s Disease Cooperative Study-activities of daily living Prevention Instrument, *TMT* Trail Making Test, *COWAT* Controlled Oral Word Association Test, *CNT* Category Naming Test, *GDS* Geriatric Depression Scale, *FCRST* Free and Cued Selective Reminding Test, *DHA* docosahexaenoic acid, *SUVr* standardized uptake value relative

### Primary objective: impact of MI on brain glucose metabolism at 6 months assessed by ROI-based approach

**Fig. 2:**
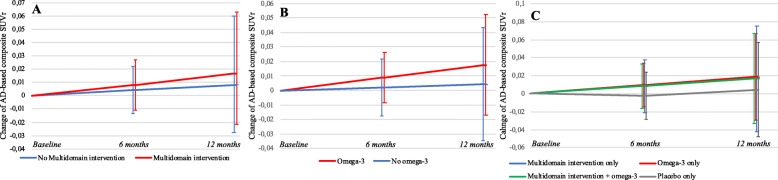
Change of AD-based SUVr from baseline to 6- and 12-month visits in the MI group and No MI group (**a**), in the Omega-3 group and No omega-3 group (**b**), and in the 3 active groups compared to control group (**c**). Abbreviations: MI, multidomain intervention; SUVr, standardized uptake value ratio

Metabolism did not increase significantly in the MI group (AD-based SUVr + 0.008, *p* = .395) and No MI group (+ 0.004, *p* = .653, Fig. 2) from baseline to 6-month visit. The comparison of change between the MI group and the No MI group showed a non-significant difference in favor of the MI group (+ 0.004, *p* = .752, Table 2).

**Table 2:** Estimated mean difference in 6- and 12-month change from baseline on brain glucose metabolism for the active groups compared to the control group

Groups		n	Estimated mean within-group change from baseline (95% CI)	Estimated mean between-group difference in change from baseline (95%CI)		
				Vs control	***p***	Adjusted p*
**Primary analysis**						
**Effect of MI** **At 6 months**	MI plus placebo or omega-3	34	0.008 (− 0.011; 0.027)	0.004 (− 0.022; 0.030)	0.752	0.901
	No MI plus placebo or omega-3	33	0.004 (− 0.014; 0.022)	–	–	–
**Secondary analysis**						
**Effect of MI at 12 months**	MI plus placebo or omega-3	34	0.016 (− 0.022; 0.055)	0.008 (− 0.044; 0.061)	0.752	0.901
	No MI plus placebo or omega-3	33	0.008 (− 0.027; 0.044)	–	–	–
**Effect of omega-3 at 6 months**	Omega-3 plus MI or no MI	38	0.009 (− 0.009; 0.026)	0.007 (− 0.019; 0.033)	0.612	0.352
	No omega-3 plus MI or no MI	29	0.002 (−0.018; 0.022)	–	–	–
**Effect of omega-3 at 12 months**	Omega-3 plus MI or no MI	38	0.018 (− 0.017; 0.052)	0.013 (− 0.039; 0.066)	0.612	0.352
	No omega-3 plus MI or no MI	29	0.004 (− 0.035; 0.043)	–	–	–
**Exploratory analysis**						
**Effect of MI, omega-3, and combination of both at 6 months**	MI plus omega-3	20	0.008 (− 0.017; 0.033)	0.010 (− 0.026; 0.047)	0.572	0.552
	Omega-3 only	18	0.009 (− 0.015; 0.033)	0.012 (− 0.024; 0.047)	0.524	0.236
	MI only	14	0.008 (− 0.021; 0.038)	0.010 (− 0.029; 0.050)	0.603	0.637
	Placebo	15	− 0.002 (− 0.028; 0.024)	–	–	–
**Effect of MI, omega-3, and combination of both at 12 months**	MI plus omega-3	20	0.017 (− 0.034; 0.067)	0.021 (− 0.052; 0.093)	0.572	0.552
	Omega-3 only	18	0.019 (− 0.030; 0.067)	0.023 (− 0.048; 0.094)	0.524	0.236
	MI only	14	0.017 (− 0.042; 0.075)	0.021 (− 0.058; 0.100)	0.603	0.637
	Placebo	15	− 0.004 (− 0.057; 0.048)	–	–	–

*Analysis adjusted for age, sex, level of education, APO ε4 genotype, clinical dementia rating global score, and group intervention

*Abbreviation*: *MI* multidomain intervention

### Secondary objectives

There was no significant increase of metabolism from baseline to 12 months in the MI group (+ 0.016, *p* = .395) and No MI group (+ 0.008, *p* = .653). No significant difference between the MI group and No MI group was observed on AD-based SUVr change from baseline to 12 months (0.008, *p* = .752, Table 2 and Fig. 2).

Metabolism increased not significantly in the Omega-3 group and No omega-3 group from baseline to 6- (respectively + 0.009 and + 0.002, *p* = .319 and *p* = .835) and 12-month visits (+ 0.018 and + 0.004, *p* = .319 and *p* = .835). No omega-3 supplementation effect was observed on AD-based SUVr change either at 6 months (+ 0.007, *p* = .612) or 12 months (+ 0.013, *p* = .612, Table 2 and Fig. 2).

### Exploratory objectives

#### Exploratory objectives assessed by ROI-based approach

From baseline to 6 and 12 months, metabolism did not increase significantly in the MI plus omega-3 group (respectively + 0.008 and + 0.017, *p* = .516), in the Omega-3 only group (+ 0.009 and + 0.019, *p* = .445), and MI only group (+ 0.008 and + 0.017, *p* = .580, Table 2 and Fig. 2). The control group was the only group with a decrease of metabolism at 6- and 12-month visits (− 0.002 and − 0.004) in AD-related brain areas (Table 2), but these differences were not significant (*p* = .871). No significant effect was found at 6 and 12 months when exploring for possible differences in metabolic change in the MI only group (0.010 and 0.021, *p* = .603), in the Omega-3 only group (0.012 and 0.023, *p* = .524), and in the MI plus omega-3 (0.010 and 0.021, *p* = .572) compared to the control group.

#### Exploratory objectives assessed by voxel-wise approach

**Fig. 3:**
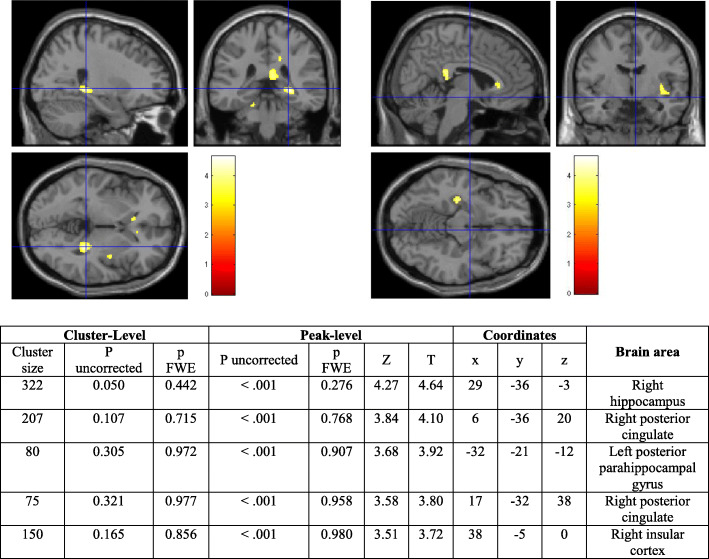
Results from the voxel-wise analysis comparing difference of metabolism from baseline to 6 months between the MI group and No MI group in MAPT-NI subjects

At 12 months, there was no significant difference on metabolism change from baseline between the MI group and No MI group. At 6 months, the MI group increased metabolism compared to the No MI group in right hippocampus, right posterior cingulate, left posterior parahippocampal gyrus, and right insular cortex (uncorrected *p* < .001, *k* > 50 voxels, Fig. 3). No difference on change of metabolism was found between Omega-3 group and No omega-3 group, either at 6 or 12 months. A difference on metabolism change was found in the right middle temporo-occipital gyrus when comparing the 4 groups (ANOVA), MI only group and omega-3 only group to control group (uncorrected *p* < .001 and *p* FEW < .05, *k* > 50 voxels, Table 3) at 6 months.

**Table 3:** Results from the voxel-wise analysis comparing difference of metabolism from baseline to 6 months between the four groups, MI only group and control group, Omega-3 only group and control group

Cluster-level			Peak-level				Coordinates			Brain area
Cluster size	*P* uncorrected	*p* FWE	*P* uncorrected	*p* FWE	*Z*	*T*	*x*	*y*	*z*	
**ANOVA**										
79	0.200	0.974	< .001	0.626	4.06	–	68	− 41	− 6	Right temporo-occipital gyrus
**Post hoc analysis**										
61	0.084	0.003	< .001	0.003	3.83	4.53	68	− 41	− 6	Right temporo-occipital gyrus
79	0.125	0.004	< .001	< .001	4.33	5.12	69	− 39	− 8	Right temporo-occipital gyrus

Maps were thresholded at *p* < 0.001 (uncorrected) and *K* > 50 voxels

*Abbreviation: FEW* family-wise error

#### Effect of adherence and impact on cognitive performances

The adherence (at least 75% of MI sessions or omega-3 supplementation) was 58.82% for the MI group and 78.94% for the Omega-3 group. Analyses of effect of MI, omega-3, and both according to adherence on metabolism were negative at 6- and 12-month visits (supplementary Table 1).

There was no significant difference between change on cognitive composite score from baseline to 6 and 12 months between the MI group and No MI group, between the Omega-3 group and No Omega group, and between the 3 active groups compared to the control group (supplementary Table 2). We only observed a marginal cognitive effect at 12 months in Omega-3 group with adherence < 75% (*n* = 8) compared to control group (*p* = .002, supplementary Table 3).

## Discussion

The hypothesis of a potential MI effect in early AD stemmed from a cognitive benefit reported in positive amyloid subjects and APOE-4 carriers included respectively in MAPT-AV45 [3] and FINGER studies [4]. We expected that MI could affect positively metabolism in regions involved early in AD. However, comparison of metabolic change from baseline to 6 and 12 months, between the MI group and the No MI group, showed no effect on the FDG-based AD biomarker. The results were similar in the analysis performed to test omega-3 supplementation and according adherence. The exploratory voxel-wise approach showed that the MI group had significant greater increase of metabolism compared to the No MI group at 6 months mainly in limbic lobe. No effect of MI was found at 12 months probably due to closer frequency of MI sessions at the beginning of MAPT-NI. An effect on metabolism of temporo-occipital gyrus was showed in the MI only group. Temporo-occipital gyrus is affected in AD and involved on visuo-spatial process [24]. This increased metabolism is possibly due to MAPT memory program based mainly on mental imaging.

MI effect on limbic system metabolism at 6 months could suggest a disease-modifying effect on AD. Indeed, glucose metabolism is known to be associated with cognitive impairment severity [25], and limbic lobe is considered as a system involved early in AD [22]. In the literature, several studies suggested the effect of lifestyle on AD-based biomarkers. In dominant autosomal AD subjects, a high physical activity was associated with a lower AD-like pathology in cerebrospinal fluid [26]. In clinical trials, physical activity increased significantly hippocampal volume in older women with mild cognitive impairment (MCI) [27] and supplementation with B vitamins slowed the rate of brain atrophy in MCI subjects [28]. A program combining cognitive and physical training increased also parahippocampal cerebral blood flow [29].

### Strengths

The strengths of MAPT-NI were the duration of intervention and objectives designed to assess the MI impact especially on FDG-based AD biomarker. We performed a ROI-based approach well described in the literature to assess the potential MI effect in early AD [22] and a complementary voxel-wise approach without anatomical hypothesis. Most trials that assessed effect of non-drug interventions on metabolism last less than 6 months [30]. The duration of MAPT-NI allowed to assess the MI effect by taking in account effect of potential associated disease progression on metabolism [30, 31]. Alexander et al. showed that brain metabolism is a sensitive marker of disease progression in AD over a 1-year period [31]. In a sub-group MRI analysis of FINGER, no differences between active and control groups were found on the changes of regional volumes and cortical thickness, while the main study was positive on cognition [32]. Glucose metabolism is likely to be a more sensitive outcome involved earlier in the hypothetical AD model than atrophy biomarkers [33].

### Limitations

The main limitation of MAPT-NI concerns the amyloid status, which was not known in MAPT-NI. Participants of MAPT-NI could not be recruited in MAPT-AV45 and reciprocally for safety reasons of radioprotection. Primary analysis could be negative potentially because only few positive amyloid subjects were included in MAPT-NI (from MAPT-AV45, we can expect approximately 30% of positive amyloid participants in MAPT-NI). In the absence of brain MRI scan at the 6-month visit, we did not perform FDG analysis in the subject MRI space but only in the MNI space which can potentially induce artifacts. Another limitation concerns the allocation of omega-3 supplementation in the MI group and No MI group. Indeed, the control group was the only group to show a decrease in glucose metabolism at 6 and 12 months while the combined intervention group (MI plus omega-3 group) did not increase brain metabolism compared to the MI only group. So, a study with larger groups of MI only and No MI without omega-3 supplementation (control group) could provide more promising results in favor of the MI only group.

## Conclusion

MI had no significant effect on FDG-based AD biomarker. However, voxel-wise analysis showed an impact on limbic lobe at 6 months suggesting delay effect on AD progression. The lack of MI effect showed at 12 months suggests the necessity to maintain high frequency of MI sessions all along interventional program. These elements will need to be investigated further in non-demented subjects with positive amyloid status. The ongoing MIND-AD study (NCT03249688) which assess the effect of a MI and medical food on cognition in prodromal AD could provide an answer such as the Dutch study NL-ENIGMA (Effect of a specific Nutritional Intervention on cerebral Glucose Metabolism in early Alzheimer’s disease) which explore effect of multinutrient combination in early AD on a FDG-based AD biomarker [34].

## Supplementary information

**Additional file 1: Supplementary Fig. 1.** Predefined cortical regions of interest included in primary outcome from Harvard-Oxford atlas. **Supplementary Table 1.** Estimated mean difference in 6- and 12-month change from baseline on brain glucose metabolism for the intervention groups according to adherence compared to the « Control group » (No MI and no omega-3 supplementation). **Supplementary Table 2.** Estimated mean difference in 6- and 12-month change from baseline on cognitive composite score for the intervention groups compared to the « Control group ». **Supplementary Table 3.** Estimated mean difference in 6- and 12-month change from baseline on cognitive composite score for the intervention groups according to adherence compared to the « Control group ».

## Data Availability

The datasets generated and/or analyzed during the current study are not publicly available. However, clinical and FDG PET data can be shared by request via “Application for Access to the MAPT Database” (for further information contact of the Data Sharing Alzheimer group: Info.u1027-dsa@inserm.fr).

## Abbreviations

AD: Alzheimer disease
DHA: Docosahexaenoic acid
EPA: Eicosapentaenoic acid
FDG: Fluorodeoxyglucose
PET: Positron emission tomography
MI: Multidomain intervention
MAPT: Multidomain Alzheimer Prevention Trial
MRI: Magnetic resonance imaging
ROI: Region of interest
SUVr: Standardized uptake value ratio
