# Supplementary material for: The impact of a multi-domain intervention on cerebral glucose metabolism: analysis from the randomized ancillary FDG PET MAPT trial
Source: Alzheimers Res Ther. 2020 Oct 19;12:134. doi: 10.1186/s13195-020-00683-6 (PMC7574215; doi:10.1186/s13195-020-00683-6)
Supplement: Supplementary file 1 — Additional file 1: Supplementary Fig. 1. Predefined cortical regions of interest included in primary outcome from Harvard-Oxford atlas. Supplementary Table 1. Estimated mean difference in 6- and 12-month change from baseline on brain glucose metabolism for the intervention groups according to adherence compared to the « Control group » (No MI and no omega-3 supplementation). Supplementary Table 2. Estimated mean difference in 6- and 12-month change from baseline on cognitive composite score for the intervention groups compared to the « Control group ». Supplementary Table 3. Estimated mean difference in 6- and 12-month change from baseline on cognitive composite score for the intervention groups according to adherence compared to the « Control group ». [file 13195_2020_683_MOESM1_ESM.docx]

**Supplementary figure 1** Predefined cortical regions of interest included in primary outcome from Harvard-Oxford atlas

**
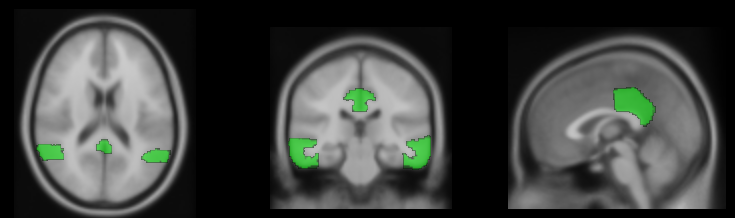
**

| **Groups** | | **n** | **Estimated mean within-group change from baseline (95% CI)** | **Estimated mean between-group difference in change from baseline (95%CI)** | | |
| --- | --- | --- | --- | --- | --- | --- |
|  |  |  |  | **Vs « control »** | **p** | **Adjusted p*** |
| **AT 6 MONTHS** | | | | | | |
| **Effect of MI according to adherence** | MI (adherence≥75%) plus placebo or omega-3 | 20 | 0.016 (-0.008 ; 0.039) | 0.012 (-0.018 ; 0.041) | 0.430 | 0.695 |
|  | MI (adherence<75%) plus placebo or omega-3 | 14 | -0.005 (-0.038 ; 0.028) | -0.009 (-0.046 ; 0.029) | 0.642 | 0.401 |
|  | No MI plus placebo or omega-3 | 33 | 0.004 (-0.014 ; 0.022) | - | - | - |
| **Effect of omega-3 according to adherence** | Omega-3 (adherence≥75%) plus MI or no MI | 30 | 0.009 (-0.011 ; 0.028) | 0.007 (-0.021 ; 0.034) | 0.635 | 0.451 |
|  | Omega-3 (adherence<75%) plus MI or no MI | 8 | 0.009 (-0.031 ; 0.048) | 0.006 (-0.038 ; 0.051) | 0.772 | 0.373 |
|  | No omega-3 plus MI or no MI | 29 | 0.002 (-0.018 ; 0.022) | - | - | - |
| **AT 12 MONTHS** | | | | | | |
| **Effect of MI according to adherence** | MI (adherence≥75%) plus placebo or omega-3 | 20 | 0.032 (-0.015 ; 0.078) | 0.023 (-0.035 ; 0.082) | 0.430 | 0.695 |
|  | MI (adherence<75%) plus placebo or omega-3 | 14 | -0.009 (-0.075 ; 0.056) | -0.018 (-0.092 ; 0.057) | 0.642 | 0.401 |
|  | No MI plus placebo or omega-3 | 33 | 0.008 (0.027 ; 0.044) | - | - | - |
| **Effect of omega-3 according to adherence** | Omega-3 (adherence≥75%) plus MI or no MI | 30 | 0.017 (-0.021 ; 0.056) | 0.013 (-0.042 ; 0.069) | 0.635 | 0.451 |
|  | Omega-3 (adherence<75%) plus MI or no MI | 8 | 0.017 (-0.062 ; 0.096) | 0.013 (-0.075 ; 0.101) | 0.772 | 0.373 |
|  | No omega-3 plus MI or no MI | 29 | 0.004 (-0.035 ; 0.043) | - | - | - |

**Supplementary table 1** Estimated mean difference in 6- and 12-month change from baseline on brain glucose metabolism for the intervention groups according to adherence compared to the « Control group » (No MI and no omega-3 supplementation)

*Analysis adjusted for age, sex, level of education, APO ε4 genotype, clinical dementia rating global score and intervention group.

MI, Multidomain Intervention.

| **Groups** | | **n** | **Estimated mean within-group change from baseline (95% CI)** | **Estimated mean between-group difference in change from baseline (95%CI)** | | |
| --- | --- | --- | --- | --- | --- | --- |
|  |  |  |  | **Vs « control »** | **p** | **Adjusted p*** |
| **AT 6 MONTHS** | | | | | | |
| **Effect of MI** | MI plus placebo or omega-3 | 34 | 0.057 (-0.123 ; 0.237) | 0.024 (-0.225 ; 0.273) | 0.849 | 0.710 |
|  | No MI plus placebo or omega-3 | 33 | 0.033 (-0.139 ; 0.206) | - | - | - |
| **Effect of omega-3** | Omega-3 plus MI or no MI | 38 | 0.138 (-0.027 ; 0.303) | 0.203 (-0.040 ; 0.446) | 0.101 | 0.335 |
|  | No omega-3 plus MI or no MI | 29 | -0.065 (-0.243 ; 0.114) | - | - | - |
| **Effect of MI, omega-3, and combination of both** | MI plus omega-3 | 20 | 0.215 (-0.016 ; 0.446) | 0.218 (0.114 ; 0.550) | 0.197 | 0.313 |
|  | Omega-3 only | 18 | 0.071 (-0.157 ; 0.299) | 0.074 (-0.256 ; 0.404) | 0.658 | 0.625 |
|  | MI only | 14 | -0.130 (-0.389 ; 0.129) | -0.127 (-0.479 ; 0.225) | 0.476 | 0.990 |
|  | Placebo | 15 | -0.003 (-0.241 ; 0.235) | - | - | - |
| **AT 12 MONTHS** | | | | | | |
| **Effect of MI** | MI plus placebo or omega-3 | 34 | 0.169 (-0.013 ; 0.352) | 0.084 (-0.165 ; 0.333) | 0.506 | 0.483 |
|  | No MI plus placebo or omega-3 | 33 | 0.085 (-0.085 ; 0.256) | - | - | - |
| **Effect of MI** | Omega-3 plus MI or no MI | 38 | 0.042 (-0.121 ; 0.205) | -0.179 (-0.424 ; 0.065) | 0.149 | 0.107 |
|  | No omega-3 plus MI or no MI | 29 | 0.221 (0.040 ; 0.403) | - | - | - |
| **Effect of MI, omega-3, and combination of both** | MI plus omega-3 | 20 | 0.087 (-0.158 ; 0.333) | -0.147 (-0.494 ; 0.200) | 0.404 | 0.446 |
|  | Omega-3 only | 18 | -0.024 (-0.242 ; 0.194) | -0.258 (-0.587 ; 0.070) | 0.122 | 0.113 |
|  | MI only | 14 | 0.216 (-0.044 ; 0.477) | -0.018 (-0.376 ; 0.340) | 0.922 | 0.984 |
|  | Placebo | 15 | 0.234 (-0.011 ; 0.479) | - | - | - |

**Supplementary table 2** Estimated mean difference in 6- and 12-month change from baseline on cognitive composite score for the intervention groups compared to the « Control group »

*Analysis adjusted for age, sex, level of education, APO ε4 genotype, clinical dementia rating global score and group intervention.

MI, Multidomain Intervention.

| **Groups** | | **n** | **Estimated mean within-group change from baseline (95% CI)** | **Estimated mean between-group difference in change from baseline (95%CI)** | | |
| --- | --- | --- | --- | --- | --- | --- |
|  |  |  |  | **Vs « control »** | **p** | **Adjusted p*** |
| **AT 6 MONTHS** | | | | | | |
| **Effect of MI according to adherence** | MI (adherence≥75%) plus placebo or omega-3 | 20 | 0.142 (-0.075 ; 0.360) | 0.109 (-0.166 ; 0.384) | 0.433 | 0.517 |
|  | MI (adherence<75%) plus placebo or omega-3 | 14 | -0.090 (-0.387 ; 0.206) | -0.124 (-0.464 ; 0.217) | 0.474 | 0.792 |
|  | No MI plus placebo or omega-3 | 33 | 0.033 (-0.135 ; 0.201) | - | - | - |
| **Effect of omega-3 according to adherence** | Omega-3 (adherence≥75%) plus MI or no MI | 30 | 0.122 (-0.047 ; 0.291) | 0.186 (-0.049 ; 0.422) | 0.120 | 0.516 |
|  | Omega-3 (adherence<75%) plus MI or no MI | 8 | 0.529 (0.082 ; 0.976) | 0.594 (0.116 ; 1.071) | 0.015 | 0.023 |
|  | No omega-3 plus MI or no MI | 29 | -0.065 (-0.232 ; 0.102) | - | - | - |
| **AT 12 MONTHS** | | | | | | |
| **Effect of MI according to adherence** | MI (adherence≥75%) plus placebo or omega-3 | 20 | 0.318 (0.096 ; 0.540) | 0.233 (-0.044 ; 0.510) | 0.099 | 0.141 |
|  | MI (adherence<75%) plus placebo or omega-3 | 14 | -0.051 (-0.360 ; 0.257) | -0.136 (-0.486 ; 0.214) | 0.443 | 0.384 |
|  | No MI plus placebo or omega-3 | 33 | 0.085 (-0.081 ; 0.251) | - | - | - |
| **Effect of omega-3 according to adherence** | Omega-3 (adherence≥75%) plus MI or no MI | 30 | 0.122 (-0.047 ; 0.291) | -0.096 (-0.336 ; 0.144) | 0.431 | 0.397 |
|  | Omega-3 (adherence<75%) plus MI or no MI | 8 | -0.217 (-0.580 ; 0.147) | -0.435 (-0.836 ; -0.034) | 0.034 | 0.002 |
|  | No omega-3 plus MI or no MI | 29 | 0.218 (0.048 ; 0.388) | - | - | - |

**Supplementary table 3** Estimated mean difference in 6- and 12-month change from baseline on cognitive composite score for the intervention groups according to adherence compared to the « Control group »

*Analysis adjusted for age, sex, level of education, APO ε4 genotype, clinical dementia rating global score and group intervention.

MI, Multidomain Intervention.
